# Supplementary figures and images for: Spatial neuronal synchronization and the waveform of oscillations: Implications for EEG and MEG
Source: PLoS Comput Biol. 2019 May 14;15(5):e1007055. doi: 10.1371/journal.pcbi.1007055 (PMC6534335; doi:10.1371/journal.pcbi.1007055)

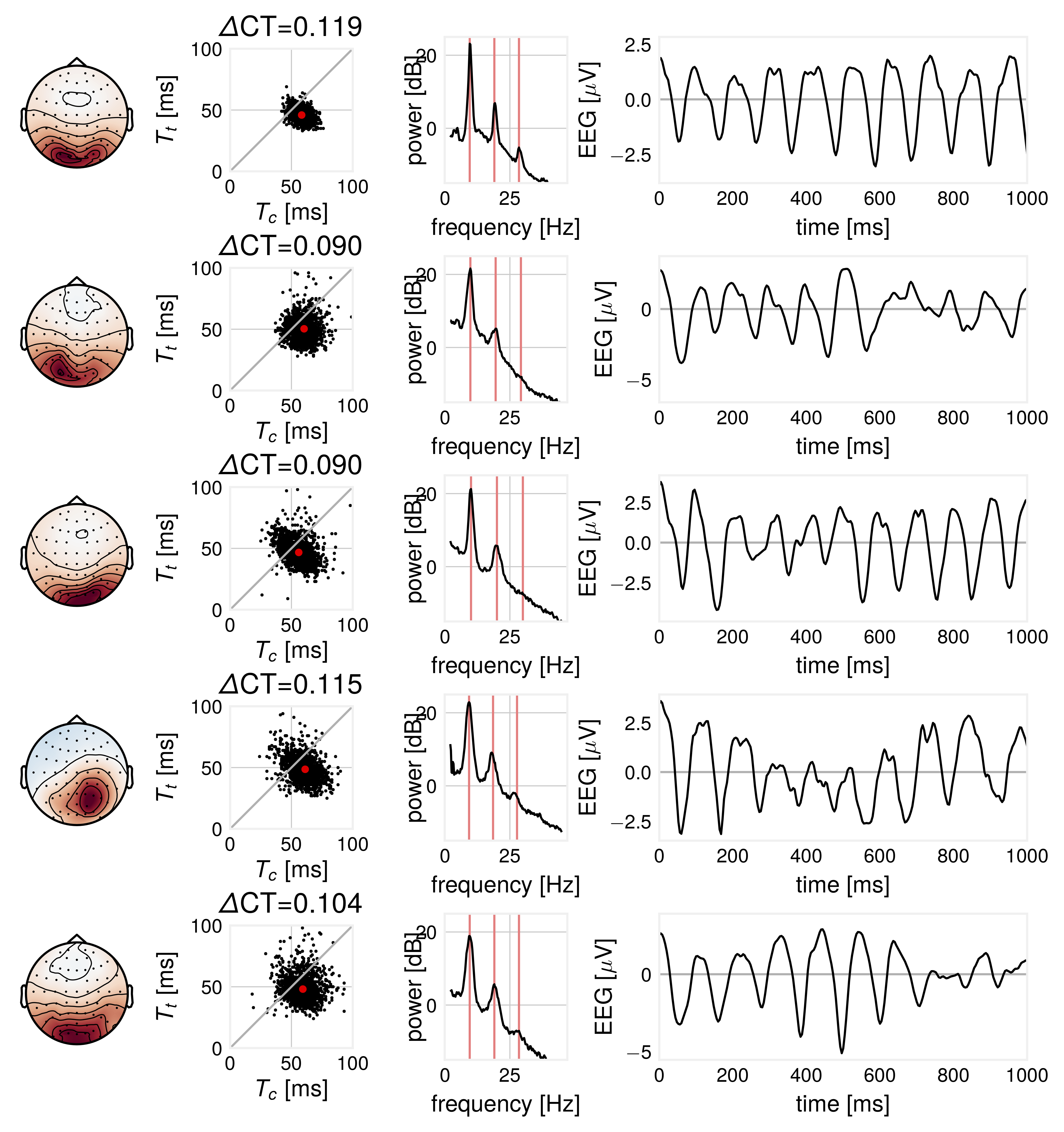

Supplement: S1 Fig — All example power spectra were computed on Laplacian-filtered C3-signals. The spectra show a clear presence of peaks in the frequency band of interest, thus justifying the selection of these subjects for further analysis. The SNR-threshold for inclusion was 5 dB. (TIFF) [file pcbi.1007055.s001.tiff]

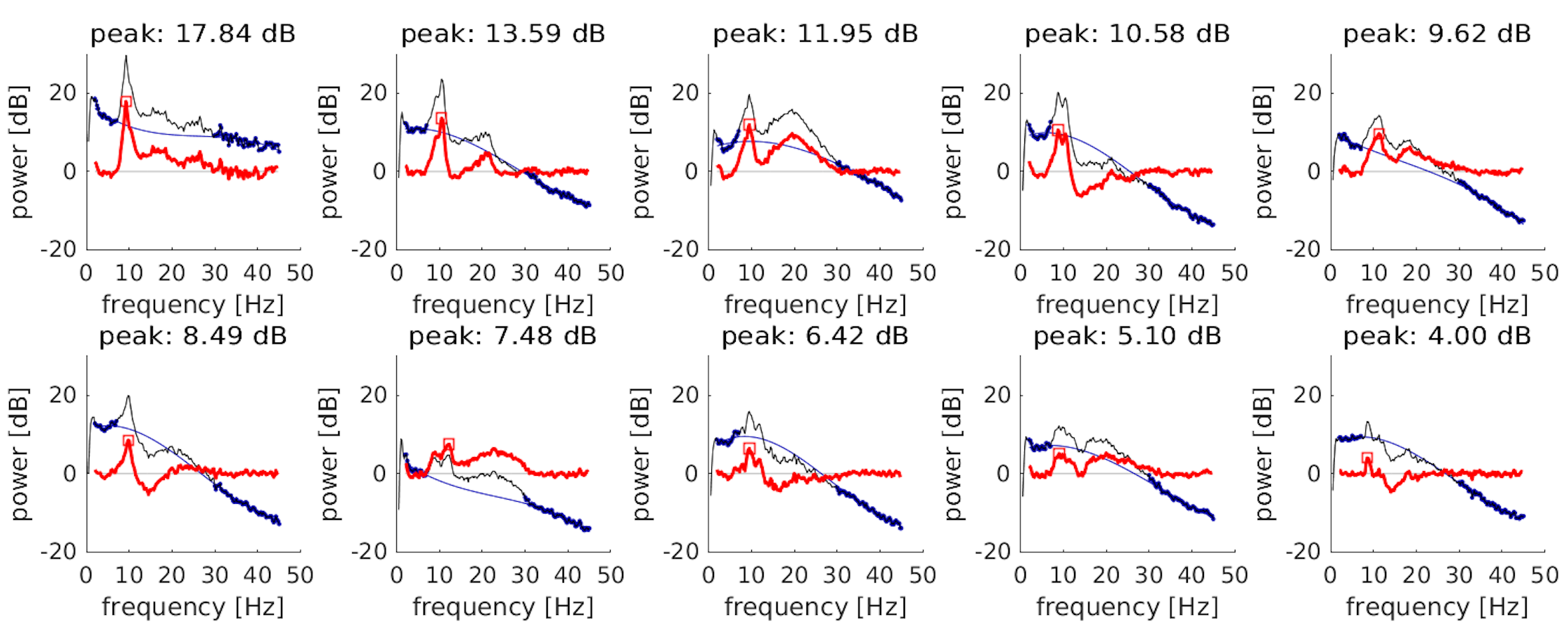

Supplement: S2 Fig — Each row corresponds to one SSD component extracted for different subjects. From left to right column: (1) Occipital topography. (2) ΔCT distributions. For every oscillatory cycle, there are corresponding Tc- and Tt-values. Red dot indicates mean Tc- and Tt-values. (3) Power spectrum showing pronounced α- and β-peaks. Pink lines indicate α-peak and first and second harmonic frequency. (4) Example time course excerpt of the SSD component. (TIFF) [file pcbi.1007055.s002.tiff]

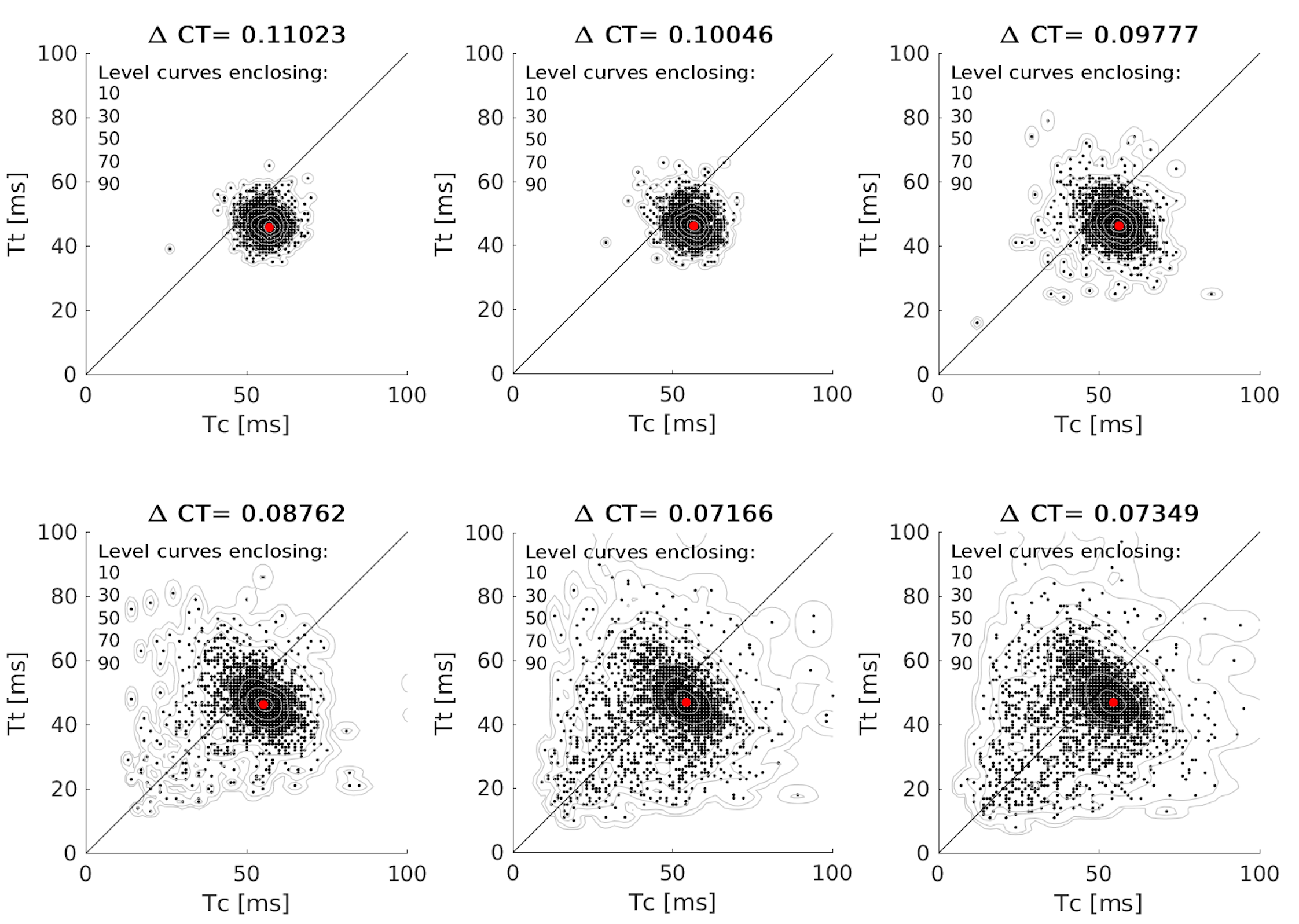

Supplement: S3 Fig — The spread of Tc- and Tt-values increases with increasing noise level. Illustration was created by adding an increasing amount of 1/f-noise to a oscillatory component as extracted by SSD. (TIFF) [file pcbi.1007055.s003.tiff]
